# CTGF increases vascular endothelial growth factor-dependent angiogenesis in human synovial fibroblasts by increasing miR-210 expression

**DOI:** 10.1038/cddis.2014.453

**Published:** 2014-10-23

**Authors:** S-C Liu, S-M Chuang, C-J Hsu, C-H Tsai, S-W Wang, C-H Tang

**Affiliations:** 1 Graduate Institute of Basic Medical Science, China Medical University, Taichung, Taiwan; 2 Institute of Biomedical Sciences, National Chung Hsing University, Taichung, Taiwan; 3 School of Chinese Medicine, College of Chinese Medicine, China Medical University, Taichung, Taiwan; 4 Department of Orthopedic Surgery, China Medical University Hospital, Taichung, Taiwan; 5 Department of Medicine, Graduate Institute of Clinical Medical Science, China Medical University, Taichung, Taiwan; 6 Department of Medicine, Mackay Medical College, New Taipei, Taiwan; 7 Department of Pharmacology, School of Medicine, China Medical University, Taichung, Taiwan; 8 Department of Biotechnology, College of Health Science, Asia University, Taichung, Taiwan

## Abstract

Connective tissue growth factor (CTGF, a.k.a. CCN2) is inflammatory mediator and abundantly expressed in osteoarthritis (OA). Angiogenesis is essential for OA progression. Here, we investigated the role of CTGF in vascular endothelial growth factor (VEGF) production and angiogenesis in OA synovial fibroblasts (OASFs). We showed that expression of CTGF and VEGF in synovial fluid were higher in OA patients than in controls. Directly applying CTGF to OASFs increased VEGF production then promoted endothelial progenitor cells tube formation and migration. CTGF induced VEGF by raising miR-210 expression via PI3K, AKT, ERK, and nuclear factor-*κ*B (NF-*κ*B)/ELK1 pathways. CTGF-mediating miR-210 upregulation repressed glycerol-3-phosphate dehydrogenase 1-like (GPD1L) expression and PHD activity and subsequently promoted hypoxia-inducible factor (HIF)-1*α*-dependent VEGF expression. Knockdown of CTGF decreased VEGF expression and abolished OASF-conditional medium-mediated angiogenesis *in vitro* as well as angiogenesis in chick chorioallantoic membrane and Matrigel-plug nude mice model *in vivo*. Taken together, our results suggest CTGF activates PI3K, AKT, ERK, and NF-*κ*B/ELK1 pathway, leading to the upregulation of miR-210, contributing to inhibit GPD1L expression and prolyl hydroxylases 2 activity, promoting HIF-1*α*-dependent VEGF expression and angiogenesis in human synovial fibroblasts.

Osteoarthritis (OA) refers to clinical syndrome of joint pain accompanied by varying degrees of functional limitation and reduced quality of life.^1^ Cause of the OA is unclear, although obesity, aging, sex, genetic factors, and injury have been associated with increased risk of OA.^2^ Development and progression of OA are now believed to involve synovial inflammation even in early stages of the disease.^3^ Biochemical mediators like cytokines, chemokines, and growth factors were found in OA synovial fibroblasts (OASFs) that affect cellular functions of knee joints. These mediators promote inflammation, cartilage degradation, and neovascularization via activation of angiogenetic factors like vascular endothelial growth factor (VEGF),^4,5^ reportedly secreted from mechanically overloaded chondrocytes^6^ and in OA joints *in vivo.*^7^ VEGF also affects chondrocytic metabolism, leading to release of matrix metalloproteinases that degrade cartilage matrix.^8^ Anti-VEGF polyclonal antibody markedly attenuated disease severity in arthritis,^9^ indicating anti-angiogenesis as novel OA treatment.

Connective tissue growth factor (CTGF, a.k.a. CCN2) is a member of the CCN family, secreted multifunctional proteins that contain high levels of cysteine. It has been proven associated with several biological functions such as fibrosis, tissue remodeling, and tumorgenesis even to OA.^10^ The mRNA expression of CTGF has been upregulated adjacent to areas of cartilage surface damage, and present in chondro-osteophytes.^11^ In animal model, CTGF overexpression in synovial lining of mouse knee joints results in reversible synovial fibrosis and cartilage damage.^12^ Both plasma and synovial fluid CTGF concentration in OA patients were correlated with radiographic severity and could be useful for monitoring progression of OA.^13^ We previously indicated CTGF enhancing IL-6 and MCP-1 expression and promoting inflammation in OASFs,^14,15^ meaning CTGF contributes to pathogenesis of OA.

The small, noncoding microRNAs (miRNAs) transcribed from DNA are 18–24 nucleotides in length, modulating targeted gene expression via either translational repression or mRNA cleavage.^16^ It is recently reported that miRNA expression was associated with well-defined clinical pathological features and disease outcomes;^17,18^ miRNAs also have been linked with OA pathogenesis, especially for expression of genes encoding catabolic factors like matrix metalloproteinases and ADAMTS.^19^ Many evidences indicated that miR-210 as angiogenic miRNA.^20, 21, 22^ In addition, overexpression of miR-210 can stimulate formation of capillary-like structures *in vitro* when cells are cultured in Matrigel.^23^ However, the exact etiological mechanism of miR-210 in angiogenesis and OA pathogenesis is largely unknown.

Angiogenesis is essential for the development, growth, and progression of OA.^24^ VEGF, a potent angiogenic factor, is pivotal in OA pathogenesis.^7^ CTGF is cited as promoting inflammatory cytokine release during OA;^12^ its role in angiogenesis is implied in many cell types,^25,26^ but its signal pathway in VEGF production and angiogenesis in synovial fibroblasts has not been extensively studied. We explored intracellular signal pathway in CTGF-induced VEGF production in OASFs and found CTGF activating PI3K, AKT, ERK, and nuclear factor-*κ*B (NF-*κ*B)/ELK1 pathway to upregulate miR-210 expression and contributing to inhibit GPD1L expression and prolyl hydroxylases 2 (PHD2) activity as well as trigger HIF-1*α*-dependent VEGF expression and angiogenesis in human OASFs.

## Results

### CTGF promotes VEGF-dependent angiogenesis in synovial fibroblasts

**Figure 1:**
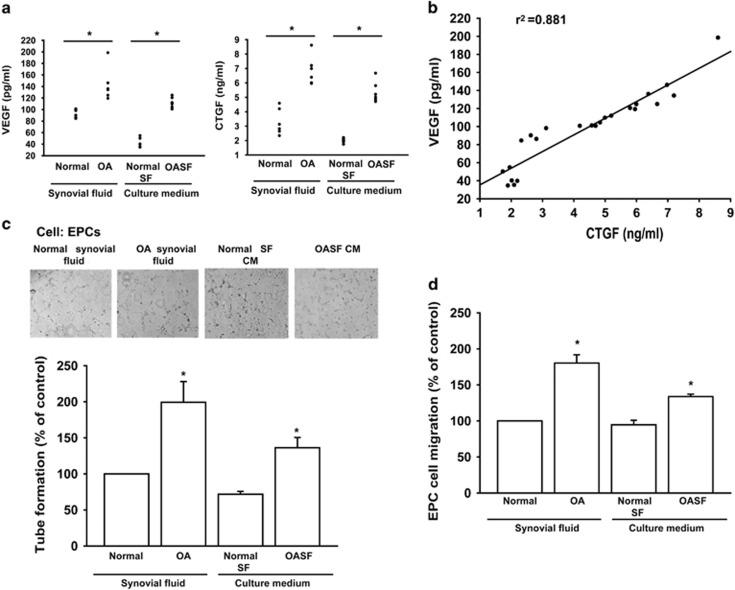
**E**xpression of CTGF and VEGF in normal and osteoarthritis (OA) patients. (**a**) Synovial fluid was obtained from normal or OA patients and conditioned medium (CM) from normal or OA synovial fibroblasts, CTGF and VEGF expression examined by ELISA. (**b**) Correlation between CTGF and VEGF expression in synovial fluid of OA patients. (**c** and **d**) Normal and OA synovial fluid or normal and OA synovial fibroblast CM were applied to EPCs for 24 h, capillary-like structure formation and migration in EPCs examined by tube formation and Transwell assay. Results are expressed as the mean±S.E.M. *P<0.05 compared with normal patients

CTGF is associated with OA pathogenesis.^12^ VEGF is most potent pro-angiogenic growth factor playing a pivotal role in angiogenesis.^4,27^ We first examined CTGF and VEGF expression in OA and normal synovial fluid to find expression of CTGF and VEGF in synovial fluid significantly higher in OA patients than in controls (Figure 1a). On the other hand, medium from OASFs exhibited significant CTGF and VEGF levels, higher than that of medium from normal SFs (Figure 1a). Expression of CTGF positively correlated with VEGF (Figure 1b). Angiogenesis mainly involves endothelial cell proliferation, migration, and tube formation to form new blood vessels.^28^ We also found OA synovial fluid or OASF conditioned medium (CM) enhancing endothelial progenitor cell (EPC) tube formation and migration (Figures 1c and d), suggesting CTGF and VEGF have key roles in OA angiogenesis and pathogenesis.

**Figure 2:**
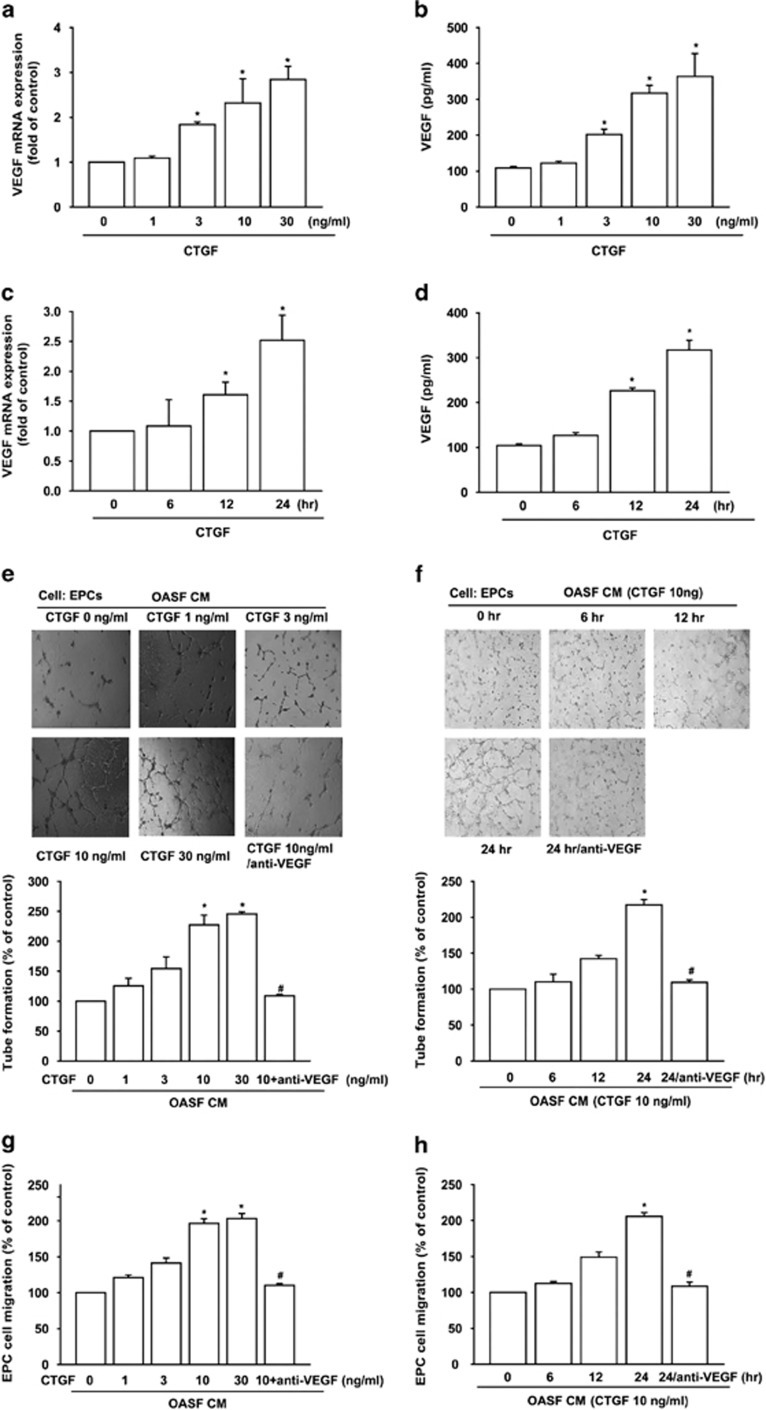
CTGF promotes VEGF-dependent angiogenesis in human synovial fibroblasts. (**a** and **b**) OASFs were incubated with various concentrations of CTGF for 24 h, VEGF expression assessed by RT-qPCR and ELISA assay. (**c** and **d**) OASFs were incubated with CTGF (10 ng/ml) for indicated time intervals, VEGF expression evaluated by RT-qPCR and ELISA assay. (**e**–**h**) OASFs pretreated for 30 min with VEGF antibody (5 μg/ml) were stimulated with CTGF (10 ng/ml) for 24 h or incubated with various concentrations of CTGF for 24 h or incubated with CTGF (10 ng/ml) for indicated time intervals. Medium collected as CM was applied to EPCs for 24 h. Cell capillary-like structure formation and migration in EPCs were examined by tube formation and Transwell assay. Results are expressed as mean±S.E.M. *P<0.05 compared with control; ^#^*P*<0.05 compared with CTGF-treated group

Next, we directly applied CTGF in OASFs and determined the expression of VEGF; CTGF raised VEGF mRNA and protein expression in a concentration- (Figures 2a and b) and time-dependent manner (Figures 2c and d). Likewise, CM from CTGF-treated OASFs promoted tube formation and migration in EPCs by concentration- and time-dependently (Figures 2e–h). To elucidate CTGF-dependent VEGF's lead role in angiogenesis, VEGF antibody was used. Figures 2e–h show pretreatment of OASFs with VEGF antibody significantly abolished CTGF-induced tube formation and migration of EPCs (Figures 2e–h), indicating CTGF-dependent VEGF expression promotes OASF angiogenesis.

### CTGF promotes VEGF production and angiogenesis by increasing miR-210 via PI3K, AKT, and ERK pathways

**Figure 3:**
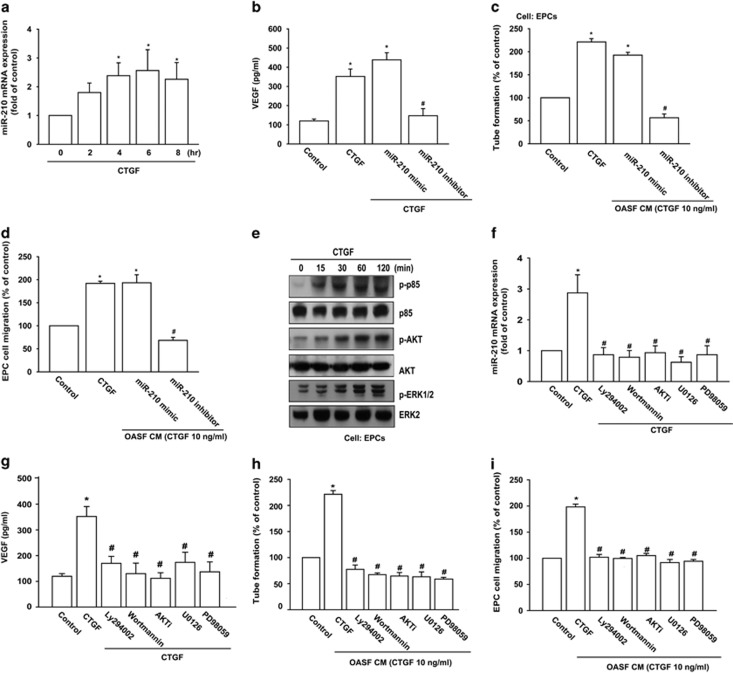
CTGF promotes VEGF production and angiogenesis through PI3K, AKT, ERK, and miR-210 pathways. (**a**) OASFs were incubated with CTGF (10 ng/ml) for indicated time intervals, miR-210 expression examined by qPCR. (**b**) OASFs were transfected with miR-210 mimic or inhibitor for 24 h followed by stimulation with CTGF for 24 h, VEGF expression rated by ELISA. (**c** and **d**) Medium collected as CM was applied to EPCs for 24 h. Capillary-like structure formation and migration in EPCs were examined by tube formation and Transwell assay. (**e**) OASFs were incubated with CTGF (10 ng/ml) for designated time intervals; P85, AKT, and ERK phosphorylation was examined by western blot analysis. (**f**) Cells were pretreated for 30 min with Ly294002 (10 *μ*M), Wortmannin (1 *μ*M), AKTi (10 *μ*M), U0126 (10 *μ*M), or PD98059 (10 *μ*M) followed by stimulation with CTGF for 24 h. MiR-210 expression was examined by qPCR. (**g**) Cells pretreated for 30 min with Ly294002, Wortmannin, AKTi, U0126, or PD98059 were stimulated with CTGF for 24 h, VEGF expression examined by ELISA. (**h** and **i**) Medium collected as CM was applied to EPCs for 24 h, capillary-like structure formation and migration in EPCs examined by tube formation and Transwell assay. Results are expressed as mean±S.E.M. *P<0.05 compared with control; ^#^*P*<0.05 compared with CTGF-treated group

Several miRNAs identified show differential expression patterns between OA and normal patients; their postulated functions relate to inflammatory, catabolic changes, and angiogenesis during OA process.^29,30^ To probe miRNA differential expression in CTGF-treated OASFs, we used a customized array of 96 human miRNAs. Three were significantly upregulated and three were downregulated (Supplementary Figure 1). Among significantly regulated miRNAs, miR-210 was most upregulated in CTGF-treated OASFs compared with controls. We directly applied CTGF in OASFs to rate miR-210 expression, which miR-210 raised in a time-dependent manner (Figure 3a). We also found miR-210 expression in OASFs sharply higher than in normal SFs (Supplementary Figure 2). To verify direct CTGF inducement of miR-210-mediating VEGF-dependent angiogenesis, we transfected miR-210 mimic or inhibitor into OASFs; miR-210 inhibitor but not mimic blocked CTGF-induced VEGF expression, EPC tube formation and migration (Figures 3b–d). These suggest miR-210 as positive regulator of CTGF-mediated, VEGF-dependent angiogenesis in OASFs.

PI3K, AKT, and ERK pathways are reported to regulate expression of miR-210 and VEGF.^31, 32, 33^ We assessed their roles in CTGF-induced miR-210 and VEGF expression. CTGF treatment of OASFs induced time-dependent phosphorylation of all three (Figure 3e). Pretreatment with PI3K (Ly294002 or Wortmannin), AKT (AKTi), and ERK inhibitors (U0126 or PD98059) for 30 min reduced CTGF-induced miR-210 expression (Figure 3f). Yet all of them suppressed CTGF-mediated VEGF production and EPC tube formation and migration (Figures 3g–i). Results portend CTGF acting via PI3K, AKT, and ERK signaling pathways to promote miR-210 expression and VEGF-dependent angiogenesis in human synovial fibroblasts.

### Involvement of NF-*κ*B and ELK1 in CTGF increases miR-210 upregulation

**Figure 4:**
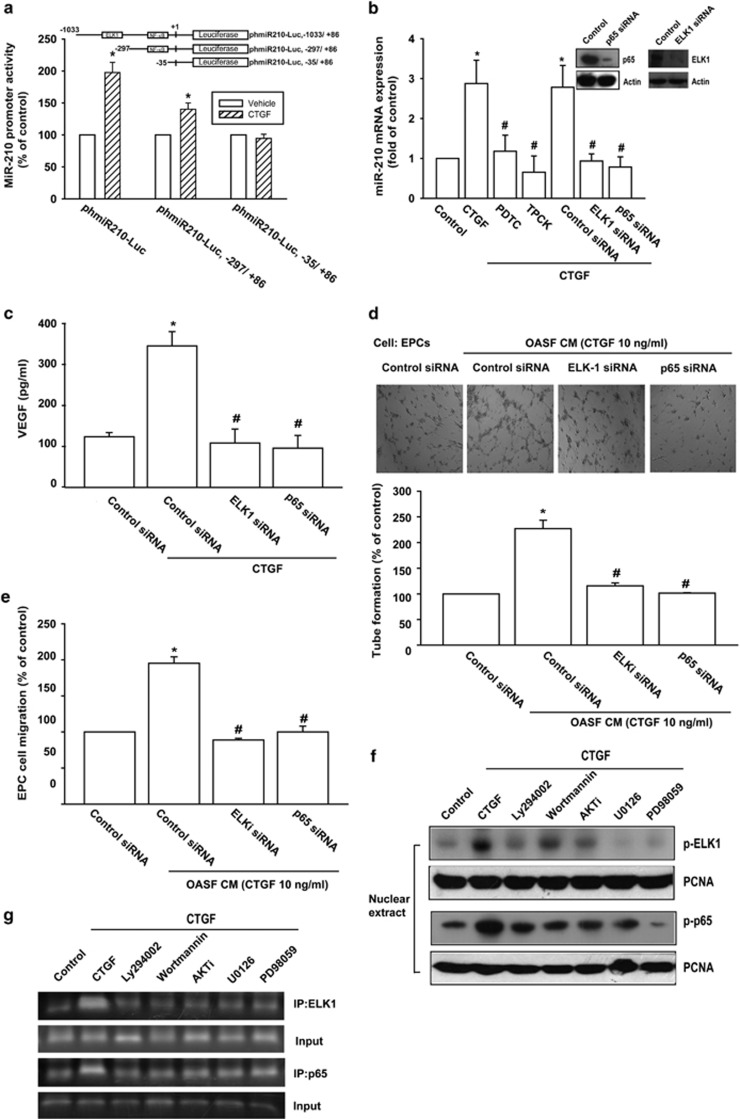
Involvement of NF-*κ*B and ELK1 in CTGF promotes miR-210 and VEGF expression as well as angiogenesis. (**a**) OASFs were transfected with miR-210 luciferase plasmids before incubation with CTGF for 24 h. Luciferase activity was then assayed. (**b**) OASFs were pretreated with PDTC (10 *μ*M) and TPCK (1 *μ*M) for 30 min or transfected with ELK1 and p65 siRNA for 24 h followed by CTGF stimulation, MiR-210 expression examined by qPCR. (**c**) OASFs were transfected with ELK1 and p65 siRNA for 24 h followed by stimulation with CTGF. VEGF expression was examined by ELISA. (**d** and **e**) Medium was collected as CM and then applied to EPCs for 24 h. Cell capillary-like structure formation and migration in EPCs were examined by tube formation and Transwell assay. (**f** and **g**) OASFs were pretreated with Ly294002, Wortmannin, AKTi, U0126, or PD98059 and stimulated with CTGF for 120 min, with p65 and ELK1 phosphorylation in nucleus or p65 and ELK1 binding to miR210 promoter determined by western blot and chromatin immunoprecipitation assay. Results are expressed as the mean±S.E.M. *P<0.05 compared with control; ^#^*P*<0.05 compared with CTGF-treated group

The miR-210 promoter contains NF-*κ*B- and ELK1-binding sites.^34^ To determine which transcription factors are involved in CTGF-induced miR-210 expression, three different length miR-210 promoter constructs were generated. Figure 4a shows that CTGF significantly increased phmiR210-Luc (−1033/+86) promoter activity, which was partially reduced in phmiR210-Luc (−297/+86; deletion ELK1-binding site) construct and completely abolished in phmiR210-Luc (−35/+86; deletion ELK1- and NF-*κ*B-binding sites) construct. The role of NF-*κ*B and ELK1 was further established using the NF-*κ*B inhibitor (PDTC) and I*κ*B protease inhibitor (TPCK) or ELK1 and p65 small interfering RNA (siRNA). Figure 4b shows all these inhibitors or siRNAs markedly reducing CTGF-induced miR-210 expression. With CTGF-mediated VEGF expression or EPC tube formation and migration reduced by transfection with ELK1 and p65 siRNA (Figures 4c–e), we postulate CTGF-increased miR-210 upregulation and angiogenesis as involving NF-*κ*B and ELK1 transactivation.

We further explored whether these signaling pathways are upstream molecules in CTGF-induced NF-*κ*B and ELK1 transcriptional activation. Pretreatment of cells with Ly294002, Wortmannin, AKTi, U0126, and PD98059 abolished CTGF-increased p-p65 and p-ELK1 accumulation into nuclei as well as p65 and ELK1 binding to NF-*κ*B and ELK1 elements on miR-210 promoter (Figures 4f and g). CTGF thus increases VEGF-dependent angiogenesis by augmenting miR-210 expression through PI3K, AKT, ERK, and NF-*κ*B/ELK1 signaling pathways in human synovial fibroblasts.

### CTGF promotes HIF-1*α*-dependent VEGF expression and angiogenesis by inhibiting GPD1L expression and PHD2 activity via upregulating miR-210

**Figure 5:**
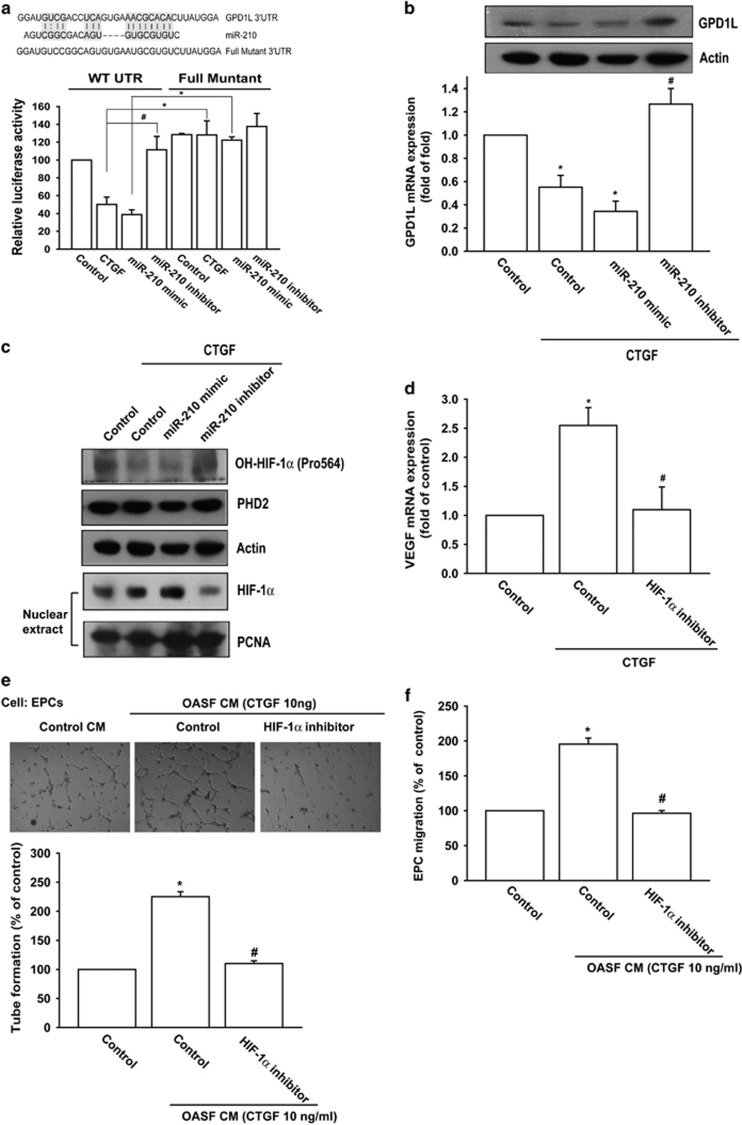
CTGF promotes HIF-1*α*-dependent VEGF expression and angiogenesis by inhibiting GPD1L expression and PHD2 hydroxylation activity by upregulation of miR-210. (**a**) Schematic 3′UTR representation of human GPD1L containing miR-210-binding site (upper panel). Cells were cotransfected with miR-210 mimic or inhibitor and wt-GPD1L-3′UTR or mt-GPD1L-3′UTR plasmid for 24 h followed by stimulation with CTGF, and relative luciferase activity was measured (lower panel). (**b**) OASFs were transfected with miR-210 mimic or inhibitor followed by CTGF stimulation, with GPD1L expression examined by RT-qPCR and western blot assay. (**c**) OASFs were transfected with miR-210 mimic or inhibitor followed by stimulation with CTGF, the expression of OH − HIF-1*α* (Pro564), PHD2, and HIF-1*α* in nucleus examined by western blot analysis. (**d**) OASFs were pretreated with HIF-1*α* inhibitor (10 *μ*M) for 30 min followed by CTGF stimulation for 24 h, VEGF expression examined by RT-qPCR assay. (**e** and **f**) Medium was collected as CM, then applied to EPCs for 24 h. Cell capillary-like structure formation and migration in EPCs were examined by tube formation and Transwell assay. Results are expressed as mean±S.E.M. *P<0.05 compared with control; ^#^*P*<0.05 compared with CTGF-treated group

To identify mechanism of miR-210-involved VEGF-dependent angiogenesis, we searched for downstream genes using bioinformative screening analysis of miRNA target databank: TargetScan, MicroCosm, and miRanda. From these three databanks overlapping between predicted targets of miR-210, GPD1L ranked as most probable target. We used luciferase reporter vectors harboring wild-type 3'-untranslated region (3′UTR) of GPD1L mRNA (wt-GPD1L-3′UTR) and vector containing mismatches in predicted miR-210-binding site (full muntant-GPD1L-3′UTR) to learn if miR-210 regulates 3′UTR of GPD1L. Figure 5a shows CTGF and miR-210 mimic inhibiting luciferase activity in wt-GPD1L-3′UTR plasmid but not in muntant-GPD1L-3′UTR plasmid. On the other hand, miR-210 inhibitor reversed CTGF-inhibited GPD1L mRNA and protein expression (Figure 5b). Therefore, miR-210 directly represses GPD1L expression through binding to 3′UTR of human *GPD1L* gene.

GPD1L is reported to potentiate activation of PHD2, further ensuring hypoxia-inducible factor 1*α* (HIF-1*α*) hydroxylation, and leading to decrease HIF-1*α* expression.^34^ It has been reported that HIF-1*α* hydroxylation at Pro-564 represents PHD2 activity.^35^ CTGF-inhibited HIF-1*α* hydroxylation was reversed by miR-210 inhibitor, leading to decrease the accumulation of HIF-1*α* into nucleus (Figure 5c), indicating CTGF-mediated miR-210 upregulation inhibits PHD2 expression and subsequently increases HIF-1*α* activation. To investigate the role of GPD1L and PHD2 in accumulation of HIF-1*α* into nucleus, the GPD1L and PHD2 siRNA were used. Transfection of GPD1L and PHD2 siRNA significantly reduced HIF-1*α* hydroxylation and increased accumulation of HIF-1*α* into nucleus (Supplementary Figure 3A). Furthermore, these siRNAs also increased EPC tube formation and migration ability (Supplementary Figures 3B and C). Next, we sought to investigate whether HIF-1*α* was involved in CTGF-induced VEGF expression in human synovial fibroblasts. We found that pretreatment with HIF-1*α* inhibitor diminished CTGF-promoted VEGF production, EPC tube formation and migration (Figures 5d–f). These suggest CTGF boosting HIF-1*α*-dependent VEGF expression and angiogenesis by inhibiting GPD1L expression and PHD2 activity through upregulation of miR-210.

### Knockdown of CTGF impairs angiogenesis *in vitro* and *in vivo*

**Figure 6:**
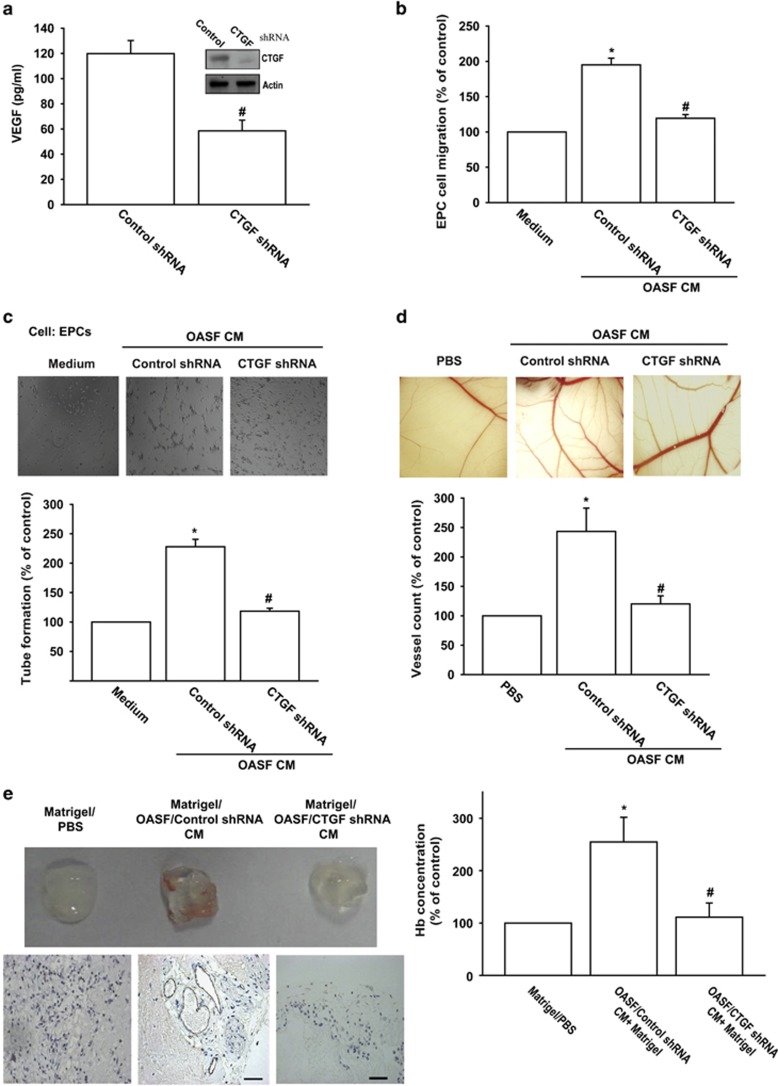
Knockdown of CTGF impairs angiogenesis *in vitro* and *in vivo.* (**a**) OASFs were transfected with control-shRNA or CTGF-shRNA, protein expression of CTGF and VEGF examined by western blot and ELISA. (**b** and **c**) Medium collected as CM was applied to EPCs for 24 h. Cell capillary-like structure formation and migration in EPCs were examined by tube formation and Transwell assay. (**d**) Chick embryos were incubated with PBS, OASFs/control-shRNA CM, or OASFs/CTGF-shRNA CM for 4 days, resected, fixed, and photographed by stereomicroscope. (**e**) Mice were injected subcutaneously with Matrigel mixed with PBS, OASFs/control-shRNA CM, or OASFs/CTGF-shRNA CM for 7 days. Plugs were excised from mice and photographed, stained with CD31, and hemoglobin content quantified. Results are expressed as mean±S.E.M. *P<0.05 compared with control; ^#^*P*<0.05 compared with CTGF-treated group

To confirm CTGF-mediated VEGF-dependent angiogenesis, CTGF-shRNA was transfected into OASFs, thus reducing CTGF and VEGF expression (Figure 6a). CTGF-shRNA also abolished OASF CM-mediated EPC tube formation and migration (Figures 6b and c). Effect of CTGF on angiogenesis *in vivo* was evaluated by *in vivo* model of chicken chorioallantoic membrane (CAM) assay. CM from OASFs increasing angiogenesis in CAM was clearly observed (Figure 6d). By contrast, knockdown of CTGF precipitously reduced angiogenesis (Figure 6d). We also analyzed Matrigel plug formation following subcutaneous implantation in mice. Matrigel mixed with CM from OASFs increased blood vessel growth (Figure 6e). Accordingly, CM from OASFs/CTGF-shRNA cells significantly curtailed neovascularization (Figure 6e). Knockdown of CTGF diminished microvessel formation in Matrigel plugs by analyzing CD31 stain and hemoglobin level (Figure 6e). Overall results indicate CTGF promoting angiogenesis *in vivo*.

## Discussion

OA is a heterogeneous group of conditions associated with defective integrity of articular cartilage as well as related changes in underlying bone. Neovascularization, formation of new blood vessels, can maintain chronic inflammatory status by transporting inflammatory cells to site of synovitis as well as supplying nutrients and oxygen to pannus.^36,37^ VEGF is a major angiogenic factor in OA joints.^38^ On the other hand, CTGF is involved in OA pathogenesis;^12,14^ its effect on VEGF expression and angiogenesis in human synovial fibroblasts is mostly unknown. Our study found expression of CTGF and VEGF in synovial fluid significantly higher than normal in OA patients, using ELISA assay. We further identified VEGF as a target protein for CTGF signaling pathway that regulates angiogenesis. Treatment of OASFs with CTGF increased VEGF expression, later inducing migration and tube formation of human EPCs. OASF CM-mediated tube formation and angiogenesis was abolished by CTGF shRNA. In addition, CTGF knockdown reduced angiogenesis *in vivo*. Our study suggests CTGF raising VEGF expression and promoting angiogenesis in human OASFs. One mechanism underlying CTGF increased VEGF yield by activating PI3K, AKT, ERK, and NF-*κ*B/ELK1 pathway, thus upregulating miR-210 expression.

The small, noncoding miRNAs regulate gene expression at post-transcriptional level by either degradation or translational repression of a target mRNA that regulates physiological and pathological processes.^39^ They and their multiple target genes reportedly play key roles in gene regulation and contribute to OA pathogenesis.^40^ It was revealed that miR-146,^41^ miR-155,^42^ and miR-203^43^ regulate arthritic inflammatory response. We screened 96 miRNAs from customized miRNA array to find miR-210 most upregulated in CTGF-treated OASFs. Also, qPCR confirmed expression of miR-210 in OASFs starkly higher than in normal SFs, indicating that miR-210 promotes angiogenesis.^44^ We saw OASF transfection with miR-210 inhibitor reducing CTGF-induced VEGF production as well as EPC tube formation and migration, making it an angiogenic gene in human OASFs. It is a direct target for ephrin-A3, protein-tyrosine phosphatase 1B, ETS domain-containing protein (ELK3), and thrombospondin that regulate angiogenesis.^45,46^ We use miRNA target prediction (TargetScan, MicroCosm, and miRanda) to find GPD1L as most probable miR-210 target, whereas CTGF decreased GPDlL mRNA expression. Transfection of miR-210 inhibitor significantly reversed CTGF-inhibited GPD1L expression. We also indicated that miR-210 directly repressed GPD1L protein expression through binding to 3′UTR of human *GPD1L* gene, thereby negatively regulating GPD1L that is documented as promoting PHD activity and later impeding HIF-1*α* expression.^34^ We proved CTGF increases accumulation of HIF-1*α* into nuclei as inhibited by miR-210 inhibitor, thus promoting HIF-1*α*-dependent VEGF expression and angiogenesis by inhibiting GPD1L expression and PHD2 activity via miR-210 upregulation.

**Figure 7:**
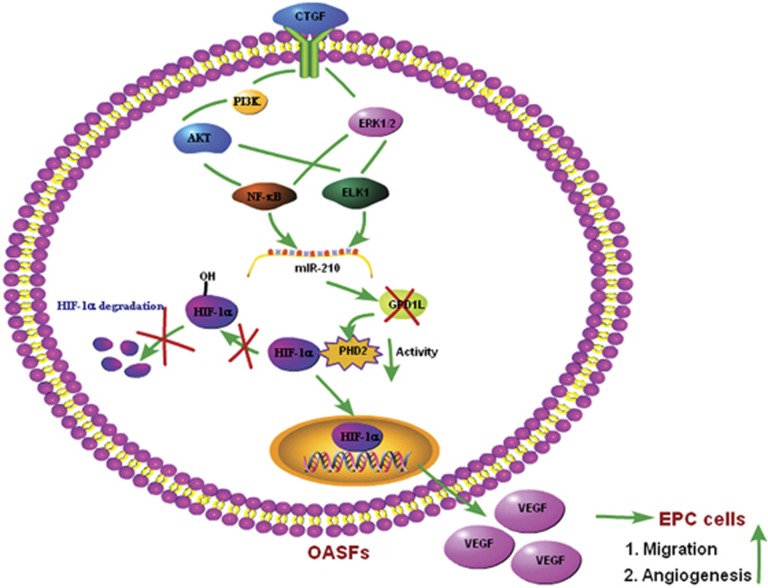
Schematic diagram of signaling pathways involved in CTGF-induced VEGF expression and angiogenesis in human synovial fibroblasts. CTGF activates PI3K, AKT, ERK, and NF-*κ*B/ELK1 pathway, leading to the upregulation of miR-210, contributing to inhibit the GPD1L expression and PHD2 activity, promoting the HIF-1*α*-dependent VEGF expression and angiogenesis in human synovial fibroblasts

PI3K, AKT, and ERK as potential candidate signaling molecules have shown capacity for regulating the VEGF expression and proliferation of endothelial cells.^47,48^ On the other hand, PI3K, AKT, and ERK reportedly regulate miR-210 expression.^33^ CTGF incubation of OASFs enhanced phosphorylation of PI3K, AKT, and ERK. Pretreatment with PI3K, AKT, or ERK inhibitors antagonized the increase of VEGF production and angiogenesis by CTGF. These inhibitors antagonized CTGF-increased miR-210 expression, indicating CTGF-increased VEGF-dependent angiogenesis via PI3K, AKT, ERK, and miR-210 signaling pathways. Angiogenesis contributes to synovitis, osteochondral damage, osteophyte formation, and meniscal pathology in OA patients. VEGF is a potent angiogenic factor pivotal in OA pathogenesis. CTGF activates PI3K, AKT, ERK, and NF-*κ*B/ELK1 pathway, leading to the upregulation of miR-210, contributing to inhibit the GPD1L expression and PHD2 activity, promoting the HIF-1*α*-dependent VEGF expression and angiogenesis in human synovial fibroblasts (Figure 7). This may lend understanding of synovial angiogenesis mechanisms to yield effective therapy.

## Materials and Methods

### Materials

Rabbit polyclonal antibodies specific to p-p85, p-AKT, p-ERK, p-p65, and p-ELK1 were purchased from Cell Signaling Technology (Danvers, MA, USA); rabbit polyclonal antibodies specific to p85, AKT, ERK, p65, ELK1, PCNA, PHD2, and the siRNAs against PHD2 and a control for experiments using targeted siRNA transfection (each consists of a scrambled sequence that does not lead to specific degradation of any known cellular mRNA) from Santa Cruz Biotechnology (Santa Cruz, CA, USA); CD31, GPD1L, and OH-HIF-1*α* (Pro564) antibody from Abcam (Cambridge, MA, USA); recombinant human CTGF and VEGF ELISA kit from PeproTech (Rocky Hill, NJ, USA); Ly294002, Wortmannin, U0126, and PD98059 from Enzo Biochem, Inc. (New York, NY, USA); miR-210 mimic, miR-210 inhibitor, Lipofectamine 2000, and Trizol from Life Technologies (Carlsbad, CA, USA). The customized miRNA array primer was obtained from System Biology Ireland (Galway, Ireland); DMEM, *α*-MEM, fetal bovine serum (FBS), and all other cell culture reagents from Gibco-BRL Life Technologies (Grand Island, NY, USA); wild-type 3′UTR of GPD1L mRNA (wt-GPD1L- 3′UTR) and vector containing mismatches in predicted miR-210-binding site (full muntant-GPD1L-3′UTR) from Addgene (Cambridge, MA); ON-TARGET*plus* siRNAs of ELK1, p65, GPD1L and control from Dharmacon Research (Lafayette, CO, USA); all other chemicals Sigma-Aldrich (St. Louis, MO, USA).

### Cell culture

Human synovial fibroblasts were isolated by collagenase treatment of synovial tissue samples obtained from patients with OA during knee replacement surgery and samples of non-arthritic synovial tissues obtained at arthroscopy after trauma/joint derangement at China Medical University Hospital. Protocol for study was approved by the Institutional Review Board at China Medical University Hospital and informed consent was obtained from each donor. OASFs were isolated, cultured, and characterized as previously described.^49^ Human endothelial progenitor cells (EPCs) were approved by the Institutional Review Board of Mackay Medical College at New Taipei City, Taiwan (reference number: P1000002). All subjects gave written consent before enrollment. Briefly, CD34-positive EPCs were maintained and propagated in MV2 complete medium consisting of MV2 basal medium and growth supplement (PromoCell, Heidelberg, Germany) of 20% FBS (HyClone, Logan, UT, USA). Cultures seeded onto 1% gelatin-coated plasticware were maintained at 37 °C in a humidified atmosphere of 5% CO_2_.^50^

### Western blot analysis

Lysates were prepared as described previously,^51^ proteins resolved by SDS-polyacrylamide gel electrophoresis and transferred to Immobilon polyvinyl difluoride membranes (Immobilon P, Millipore, Billerica, MA, USA). Blots blocked with 4% BSA for 1 h at room temperature were probed with rabbit anti-human antibodies against PI3K, AKT, ERK, p65, and ELK1 (1 : 1000) for 1 h at room temperature. After third wash, blots were incubated for 1 h at room temperature with donkey anti-rabbit peroxidase-conjugated secondary antibody (1 : 3000), then visualized by enhanced chemiluminescence with Kodak X-OMAT LS film (Eastman Kodak, Rochester, NY, USA).

### Real-time quantitative PCR (RT-qPCR) of mRNA and miRNA

Total RNA was extracted from human synovial fibroblasts by TRIzol; reverse transcription used 1 *μ*g of total RNA transcribed into cDNA by oligo (dT) primers.^49^ RT-qPCR used Taqman One-Step RT-PCR Master Mix (Applied Biosystems, Foster City, CA, USA): 2 *μ*l of cDNA template added to each 25-*μ*l reaction with Taqman probes and sequence-specific primers. Sequences for target gene primers and probes were purchased commercially. Glyceraldehyde 3-phosphate dehydrogenase served as an endogenous control to normalize expression data (Applied Biosystems), qPCR assays carried out in triplicate in a StepOnePlus sequence detection system(Applied Biosystems). The cycling conditions: initial 10-min polymerase activation at 95 °C, followed by 40 cycles at 95 °C for 15 s and 60 °C for 60 s, threshold set above non-template control background and within linear phase of target gene amplification to calculate cycle number at which transcript was detected (denoted CT). For miRNA assay, cDNA was synthesized from total RNA (100 ng) by TaqMan MicroRNA Reverse Transcription Kit (Applied Biosystems). Reactants were incubated at 16 °C for 30 min, then 42 °C for 30 min, followed by inactivation at 85 °C for 5 min. Reactions were then incubated in a 96-well plate at 50 °C for 2 min, 95 °C for 10 min, followed by 30 cycles of 95 °C for 15 s and 60 °C for 60 s with StepOnePlus sequence detection system. Relative gene expression quantification used endogenous control gene (U6). Threshold cycle (CT) was defined as fractional cycle number at which fluorescence passed a fixed threshold, relative expression calculated by comparative CT method.

### Measurements of VEGF yield

Human synovial fibroblasts were cultured in 24-well culture plates. After reaching confluence, cells were treated with CTGF, then incubated in humidified incubator at 37 °C for 24 h. To examine downstream signaling pathways involved in CTGF treatment, cells were pretreated with various inhibitors for 30 min before CTGF (10 ng/ml) administration. After incubation, medium was removed and stored at −80 °C until VEGF (in medium) was assayed by VEGF enzyme immunoassay kit, according to the procedure described by the manufacturer.

### Plasmids and constructs

Plasmid pGL2-Basic (Promega, Madison, WI, USA) was used to generate constructs of the human miR-210 (hmiR-210) promoter. To obtain phmiR-210 (phmiR210-Luc), which covered positions −1033 to +86, DNA fragments were amplified by PCR with chemically synthesized oligonucleotides that corresponded to nucleotides −1033 to −1015 of the sense strand 5′-GGAGATCTAAGACCCCTGAGCCCTTG-3′, and +68 to +86 of the antisense strand 5′-CGGCTAGCCGCTGTCACACGCACAGT-3′. Human genomic DNA derived from a healthy adult was used as the template. The PCR products were digested with *Nhe*I and *Bgl*II and cloned into the NheI-BglII site of pGL2-Basic. The phmiR-210 deletion ELK1-binding site (phmiR210-Luc, −297/+86) or both deletion ELK1- and NF-*κ*B-binding site construct (phmiR-210-Luc, −35/+86) all made by using the sense PCR primers (5′-GGAGATCTCTGAGGGACCAGGTCATTTG-3′ and 5′-GGAGATCTCAGTGCAATGATGAAAGGGCAT-3′, respectively). All the sense primers have a BglII site at 5′ end of the primer. The PCR products were digested with *Nhe*I and *Bgl*II and cloned into the NheI-BglII site of pGL2-Basic.

### Chromatin immunoprecipitation

This proceeded as earlier detailed:^52^ DNA immunoprecipitated with anti-p65 or ELK1 Ab purified and extracted into pellets with phenol-chloroform, then subjected to PCR, products resolved by 1.5% agarose gel electrophoresis and visualized with UV light. Primers 5′-ACGCGGGTGCGGGGCACGGAGGC-3′ and 5′-CGGATGGTACGGCCCGAGGTGG-3′ served to amplify across human miR-210 promoter region contain ELK1-binding site (−595 to −582), primers 5′-GGGCCGGGGGGCGAGAGGGTGCCA-3′ and 5′-TCCCCTCCAACTTGGGCGTCCGAG-3′ to amplify across human miR-210 promoter region containing ELK1-binding site (−123 to −113).

### Preparing CM

Human synovial fibroblasts plated in six-well dishes were grown to confluence, culture medium exchanged with serum-free DMEM medium. CM were collected 2 days after change of media and stored at −20 °C until use. For serial experiments, cells were pretreated for 30 min with inhibitors, including Ly294002, Wortmannin, AKTi, U0126, PD98059, and HIF-1*α* inhibitor or transfected with miR-210 mimic, miR-210 inhibitor, ELK1 siRNA, p65 siRNA, and CTGF shRNA for 24 h, followed by treatment with CTGF for 24 h to prevent signaling via CTGF pathway.

### EPCs migration assay

Migration activity was measured by Transwell assay (Corning, Costar, Tewksbury, MA, USA; pore size, 8-*μ*m). About 1 × 10^4^ EPCs were added to upper chamber in 200 *μ*l of 10% FBS MV2 complete medium. Lower chamber contained 150 *μ*l 20% FBS MV2 complete medium and 150 *μ*l CM. Plates were incubated for 24 h at 37 °C in 5% CO_2_, cells fixed in 3.7% formaldehyde solution for 15 min and stained with 0.05% crystal violet in PBS for 15 min. Cells on upper side of filters were removed with cotton-tipped swabs, filters washed with PBS. Cells on undersides of filters were examined and counted under microscope. Each clone was plated in triplicate for each experiment, each experiment repeated at least three times.

### Tube formation assay

Matrigel (BD Biosciences, Bedford, MA, USA) was dissolved overnight at 4 °C and 48-well plates prepared with 100 μl Matrigel in each well, after coating and incubating at 37 °C overnight. EPCs (3 × 10^4^) were cultured in 200 *μ*l media consisting of 50% EGM-MV2 medium and 50% CM. After 6 h incubation at 37 °C, tube formation was assessed with photomicroscope, each well photographed at × 200 magnification. Tube branches and total tube length were calculated using MacBiophotonics Image J software (Bethesda, MD, USA).

### CAM assay

Fertilized chicken eggs were incubated at 38 °C with 80% humidity. Small window was made in shell on day 3 of chick embryo development under aseptic conditions, then resealed with adhesive tape. Eggs were returned to incubator until day 3 of chick embryo development. On day 7, CM from OASFs deposited in center of *CAM*. At 11 days, *CAM*s were collected for microscopy and photographic documentation, angiogenesis quantified by counting number of blood vessel branches; at least 10 viable embryos were tested for each treatment. All animal work conformed with protocol approved by the China Medical University (Taichung, Taiwan) Institutional Animal Care and Use Committees.

### Matrigel plug assay

Matrigel plug angiogenesis was adapted from previously described assay.^53^ Four-week-old male nude mice were randomized as serum-free medium, OASFs/control-shRNA CM, or OASFs/CTGF-shRNA CM resuspended with Matrigel. Mice were subcutaneously injected with 300 *μ*l of Matrigel. After 7 days, Matrigel pellets were harvested, partially fixed with 4% formalin, embedded in paraffin, then processed for immunohistochemistry staining for vessel marker CD31.

### Statistical analysis

Data were presented as mean±standard error of the mean. Comparison between samples was performed by Student's *t-*test; for more than two groups, we used one-way analysis of variance with Bonferroni's *post-hoc* test. In all cases, *P*<0.05 was considered significant.

## Abbreviations

CTGF: Connective tissue growth factor
OA: Osteoarthritis
OASF: OA synovial fibroblast
NF-*κ*B: Nuclear factor kappa B
VEGF: vascular endothelial growth factor
MiRNAs: the small, non-coding microRNAs
3′-UTR: 3′-untranslated region
GPD1L: glycerol-3-phosphate dehydrogenase 1-like
PHD2: prolyl hydroxylases 2
CM: Conditioned medium
HIF: hypoxia-inducible factor
siRNA: small interfering RNA
EPC: endothelial progenitor cell

## Supplementary Material

Supplrmrntary Figure S1

## References

[bib1] 1Peat G, McCarney R, Croft P. Knee pain and osteoarthritis in older adults: a review of community burden and current use of primary health care. Ann Rheum Dis 2001; 60: 91–97. 11156538 10.1136/ard.60.2.91 PMC1753462

[bib2] 2Goldring MB, Goldring SR. Osteoarthritis. J Cell Physiol 2007; 213: 626–634. 17786965 10.1002/jcp.21258

[bib3] 3Felson DT. Clinical practice. Osteoarthritis of the knee. N Engl J Med 2006; 354: 841–848. 16495396 10.1056/NEJMcp051726

[bib4] 4Sellam J, Berenbaum F. The role of synovitis in pathophysiology and clinical symptoms of osteoarthritis. Nat Rev Rheumatol 2010; 6: 625–635. 20924410 10.1038/nrrheum.2010.159

[bib5] 5Ashraf S, Walsh DA. Angiogenesis in osteoarthritis. Curr Opin Rheumatol 2008; 20: 573–580. 18698180 10.1097/BOR.0b013e3283103d12

[bib6] 6Pufe T, Lemke A, Kurz B, Petersen W, Tillmann B, Grodzinsky AJ et al. Mechanical overload induces VEGF in cartilage discs *via* hypoxia-inducible factor. Am J Pathol 2004; 164: 185–192. 14695332 10.1016/S0002-9440(10)63109-4 PMC1602231

[bib7] 7Jansen H, Meffert RH, Birkenfeld F, Petersen W, Pufe T. Detection of vascular endothelial growth factor (VEGF) in moderate osteoarthritis in a rabbit model. Ann Anat 2012; 194: 452–456. 22429866 10.1016/j.aanat.2012.01.006

[bib8] 8Murata M, Yudoh K, Masuko K. The potential role of vascular endothelial growth factor (VEGF) in cartilage: how the angiogenic factor could be involved in the pathogenesis of osteoarthritis? Osteoarthr Cartil 2008; 16: 279–286. 17945514 10.1016/j.joca.2007.09.003

[bib9] 9Sone H, Kawakami Y, Sakauchi M, Nakamura Y, Takahashi A, Shimano H et al. Neutralization of vascular endothelial growth factor prevents collagen-induced arthritis and ameliorates established disease in mice. Biochem Biophys Res Commun 2001; 281: 562–568. 11181084 10.1006/bbrc.2001.4395

[bib10] 10Perbal B. CCN proteins: multifunctional signalling regulators. Lancet 2004; 363: 62–64. 14723997 10.1016/S0140-6736(03)15172-0

[bib11] 11Omoto S, Nishida K, Yamaai Y, Shibahara M, Nishida T, Doi T et al. Expression and localization of connective tissue growth factor (CTGF/Hcs24/CCN2) in osteoarthritic cartilage. Osteoarthr Cartil 2004; 12: 771–778. 15450526 10.1016/j.joca.2004.06.009

[bib12] 12Blaney Davidson EN, Vitters EL, Mooren FM, Oliver N, Berg WB, van der Kraan PM. Connective tissue growth factor/CCN2 overexpression in mouse synovial lining results in transient fibrosis and cartilage damage. Arthritis Rheum 2006; 54: 1653–1661. 16646035 10.1002/art.21795

[bib13] 13Honsawek S, Yuktanandana P, Tanavalee A, Chirathaworn C, Anomasiri W, Udomsinprasert W et al. Plasma and synovial fluid connective tissue growth factor levels are correlated with disease severity in patients with knee osteoarthritis. Biomarkers 2012; 17: 303–308. 22416876 10.3109/1354750X.2012.666676

[bib14] 14Liu SC, Hsu CJ, Chen HT, Tsou HK, Chuang SM, Tang CH. CTGF increases IL-6 expression in human synovial fibroblasts through integrin-dependent signaling pathway. PLoS One 2012; 7: e51097. 23227240 10.1371/journal.pone.0051097 PMC3515445

[bib15] 15Liu SC, Hsu CJ, Fong YC, Chuang SM, Tang CH. CTGF induces monocyte chemoattractant protein-1 expression to enhance monocyte migration in human synovial fibroblasts. Biochim Biophys Acta 2013; 1833: 1114–1124. 23274856 10.1016/j.bbamcr.2012.12.014

[bib16] 16Ambros V. The functions of animal microRNAs. Nature 2004; 431: 350–355. 15372042 10.1038/nature02871

[bib17] 17Calin GA, Croce CM. MicroRNA signatures in human cancers. Nat Rev Cancer 2006; 6: 857–866. 17060945 10.1038/nrc1997

[bib18] 18Chan WL, Chan YS, Yang WK, Huang HD, Chang JG. Very long non-coding RNA and human disease. BioMedicine 2012; 2: 167–173.

[bib19] 19Miyaki S, Asahara H. Macro view of microRNA function in osteoarthritis. Nat Rev Rheumatol 2012; 8: 543–552. 22890245 10.1038/nrrheum.2012.128 PMC3572197

[bib20] 20van Solingen C, Seghers L, Bijkerk R, Duijs JM, Roeten MK, van Oeveren-Rietdijk AM et al. Antagomir-mediated silencing of endothelial cell specific microRNA-126 impairs ischemia-induced angiogenesis. J Cell Mol Med 2009; 13: 1577–1585. 19120690 10.1111/j.1582-4934.2008.00613.x PMC3828868

[bib21] 21Chen Y, Gorski DH. Regulation of angiogenesis through a microRNA (miR-130a) that down-regulates antiangiogenic homeobox genes GAX and HOXA5. Blood 2008; 111: 1217–1226. 17957028 10.1182/blood-2007-07-104133 PMC2214763

[bib22] 22Suarez Y, Fernandez-Hernando C, Pober JS, Sessa WC. Dicer dependent microRNAs regulate gene expression and functions in human endothelial cells. Circ Res 2007; 100: 1164–1173. 17379831 10.1161/01.RES.0000265065.26744.17

[bib23] 23Fasanaro P, D'Alessandra Y, Di Stefano V, Melchionna R, Romani S, Pompilio G et al. MicroRNA-210 modulates endothelial cell response to hypoxia and inhibits the receptor tyrosine kinase ligand Ephrin-A3. J Biol Chem 2008; 283: 15878–15883. 18417479 10.1074/jbc.M800731200 PMC3259646

[bib24] 24Mapp PI, Walsh DA. Mechanisms and targets of angiogenesis and nerve growth in osteoarthritis. Nat Rev Rheumatol 2012; 8: 390–398. 22641138 10.1038/nrrheum.2012.80

[bib25] 25Brigstock DR. Regulation of angiogenesis and endothelial cell function by connective tissue growth factor (CTGF) and cysteine-rich 61 (CYR61). Angiogenesis 2002; 5: 153–165. 12831056 10.1023/a:1023823803510

[bib26] 26Yang F, Tuxhorn JA, Ressler SJ, McAlhany SJ, Dang TD, Rowley DR. Stromal expression of connective tissue growth factor promotes angiogenesis and prostate cancer tumorigenesis. Cancer Res 2005; 65: 8887–8895. 16204060 10.1158/0008-5472.CAN-05-1702

[bib27] 27Bonnet CS, Walsh DA. Osteoarthritis, angiogenesis and inflammation. Rheumatology (Oxford) 2005; 44: 7–16. 15292527 10.1093/rheumatology/keh344

[bib28] 28Carmeliet P, Jain RK. Angiogenesis in cancer and other diseases. Nature 2000; 407: 249–257. 11001068 10.1038/35025220

[bib29] 29Nakasa T, Nagata Y, Yamasaki K, Ochi M. A mini-review: microRNA in arthritis. Physiol Genomics 2011; 43: 566–570. 21325061 10.1152/physiolgenomics.00142.2010

[bib30] 30Iliopoulos D, Malizos KN, Oikonomou P, Tsezou A. Integrative microRNA and proteomic approaches identify novel osteoarthritis genes and their collaborative metabolic and inflammatory networks. PLoS One 2008; 3: e3740. 19011694 10.1371/journal.pone.0003740 PMC2582945

[bib31] 31Zhou M, Song X, Huang Y, Wei L, Li Z, You Q et al. Wogonin inhibits H2O2-induced angiogenesis *via* suppressing PI3K/Akt/NF-kappaB signaling pathway. Vascul Pharmacol 2014; 60: 110–119. 24534483 10.1016/j.vph.2014.01.010

[bib32] 32Kim MK, Park HJ, Kim YD, Ryu MH, Takata T, Bae SK et al. Hinokitiol increases the angiogenic potential of dental pulp cells through ERK and p38MAPK activation and hypoxia-inducible factor-1alpha (HIF-1alpha) upregulation. Arch Oral Biol 2014; 59: 102–110. 24370180 10.1016/j.archoralbio.2013.10.009

[bib33] 33Kim JH, Park SG, Song SY, Kim JK, Sung JH. Reactive oxygen species-responsive miR-210 regulates proliferation and migration of adipose-derived stem cells *via* PTPN2. Cell Death Dis 2013; 4: e588. 23579275 10.1038/cddis.2013.117 PMC3641340

[bib34] 34Kelly TJ, Souza AL, Clish CB, Puigserver P. A hypoxia-induced positive feedback loop promotes hypoxia-inducible factor 1alpha stability through miR-210 suppression of glycerol-3-phosphate dehydrogenase 1-like. Mol Cell Biol 2011; 31: 2696–2706. 21555452 10.1128/MCB.01242-10 PMC3133367

[bib35] 35Jaakkola P, Mole DR, Tian YM, Wilson MI, Gielbert J, Gaskell SJ et al. Targeting of HIF-alpha to the von Hippel-Lindau ubiquitylation complex by O2-regulated prolyl hydroxylation. Science 2001; 292: 468–472. 11292861 10.1126/science.1059796

[bib36] 36Maa MC, Leu TH. Activation of Toll-like receptors induces macrophage migration *via* the iNOS/Src/FAK pathway. BioMedicine 2011; 1: 11–15.

[bib37] 37Haywood L, McWilliams DF, Pearson CI, Gill SE, Ganesan A, Wilson D et al. Inflammation and angiogenesis in osteoarthritis. Arthritis Rheum 2003; 48: 2173–2177. 12905470 10.1002/art.11094

[bib38] 38Ashraf S, Mapp PI, Walsh DA. Contributions of angiogenesis to inflammation, joint damage, and pain in a rat model of osteoarthritis. Arthritis Rheum 2011; 63: 2700–2710. 21538326 10.1002/art.30422

[bib39] 39He L, Hannon GJ. MicroRNAs: small RNAs with a big role in gene regulation. Nat Rev Genet 2004; 5: 522–531. 15211354 10.1038/nrg1379

[bib40] 40Yu C, Chen WP, Wang XH. MicroRNA in osteoarthritis. J Int Med Res 2011; 39: 1–9. 21672302 10.1177/147323001103900101

[bib41] 41Taganov KD, Boldin MP, Chang KJ, Baltimore D. NF-kappaB-dependent induction of microRNA miR-146, an inhibitor targeted to signaling proteins of innate immune responses. Proc Natl Acad Sci USA 2006; 103: 12481–12486. 16885212 10.1073/pnas.0605298103 PMC1567904

[bib42] 42Stanczyk J, Pedrioli DM, Brentano F, Sanchez-Pernaute O, Kolling C, Gay RE et al. Altered expression of MicroRNA in synovial fibroblasts and synovial tissue in rheumatoid arthritis. Arthritis Rheum 2008; 58: 1001–1009. 18383392 10.1002/art.23386

[bib43] 43Stanczyk J, Ospelt C, Karouzakis E, Filer A, Raza K, Kolling C et al. Altered expression of microRNA-203 in rheumatoid arthritis synovial fibroblasts and its role in fibroblast activation. Arthritis Rheum 2011; 63: 373–381. 21279994 10.1002/art.30115 PMC3116142

[bib44] 44Liu F, Lou YL, Wu J, Ruan QF, Xie A, Guo F et al. Upregulation of microRNA-210 regulates renal angiogenesis mediated by activation of VEGF signaling pathway under ischemia/perfusion injury *in vivo* and *in vitro*. Kidney Blood Press Res 2012; 35: 182–191. 22123256 10.1159/000331054

[bib45] 45Chan SY, Loscalzo J. MicroRNA-210: a unique and pleiotropic hypoxamir. Cell Cycle 2010; 9: 1072–1083. 20237418 10.4161/cc.9.6.11006 PMC2912143

[bib46] 46Chan YC, Banerjee J, Choi SY, Sen CK. miR-210: the master hypoxamir. Microcirculation 2012; 19: 215–223. 22171547 10.1111/j.1549-8719.2011.00154.x PMC3399423

[bib47] 47Fukuda R, Hirota K, Fan F, Jung YD, Ellis LM, Semenza GL. Insulin-like growth factor 1 induces hypoxia-inducible factor 1-mediated vascular endothelial growth factor expression, which is dependent on MAP kinase and phosphatidylinositol 3-kinase signaling in colon cancer cells. J Biol Chem 2002; 277: 38205–38211. 12149254 10.1074/jbc.M203781200

[bib48] 48Prasadam I, Zhou Y, Du Z, Chen J, Crawford R, Xiao Y. Osteocyte-induced angiogenesis *via* VEGF-MAPK-dependent pathways in endothelial cells. Mol Cell Biochem 2014; 386: 15–25. 24162672 10.1007/s11010-013-1840-2

[bib49] 49Tang CH, Hsu CJ, Fong YC. The CCL5/CCR5 axis promotes interleukin-6 production in human synovial fibroblasts. Arthritis Rheum 2010; 62: 3615–3624. 20862675 10.1002/art.27755

[bib50] 50Wu MH, Huang CY, Lin JA, Wang SW, Peng CY, Cheng HC et al. Endothelin-1 promotes vascular endothelial growth factor-dependent angiogenesis in human chondrosarcoma cells. Oncogene 2014; 33: 1725–1735. 23584483 10.1038/onc.2013.109

[bib51] 51Tseng CP, Huang CL, Huang CH, Cheng JC, Stern A, Tseng CH et al. Disabled-2 small interfering RNA modulates cellular adhesive function and MAPK activity during megakaryocytic differentiation of K562 cells. FEBS Lett 2003; 541: 21–27. 12706813 10.1016/s0014-5793(03)00281-3

[bib52] 52Chiu YC, Lin CY, Chen CP, Huang KC, Tong KM, Tzeng CY et al. Peptidoglycan enhances IL-6 production in human synovial fibroblasts *via* TLR2 receptor, focal adhesion kinase, Akt, and AP-1- dependent pathway. J Immunol 2009; 183: 2785–2792. 19635908 10.4049/jimmunol.0802826

[bib53] 53Passaniti A, Taylor RM, Pili R, Guo Y, Long PV, Haney JA et al. A simple, quantitative method for assessing angiogenesis and antiangiogenic agents using reconstituted basement membrane, heparin, and fibroblast growth factor. Lab Invest 1992; 67: 519–528. 1279270

