# Supplementary material for: CTGF increases vascular endothelial growth factor-dependent angiogenesis in human synovial fibroblasts by increasing miR-210 expression
Source: Cell Death Dis. 2014 Oct 23;5(10):e1485–. doi: 10.1038/cddis.2014.453 (PMC4649533; doi:10.1038/cddis.2014.453)
Supplement: Supplrmrntary Figure S1 [file cddis2014453x1.doc]

**Supplemental Data**

**
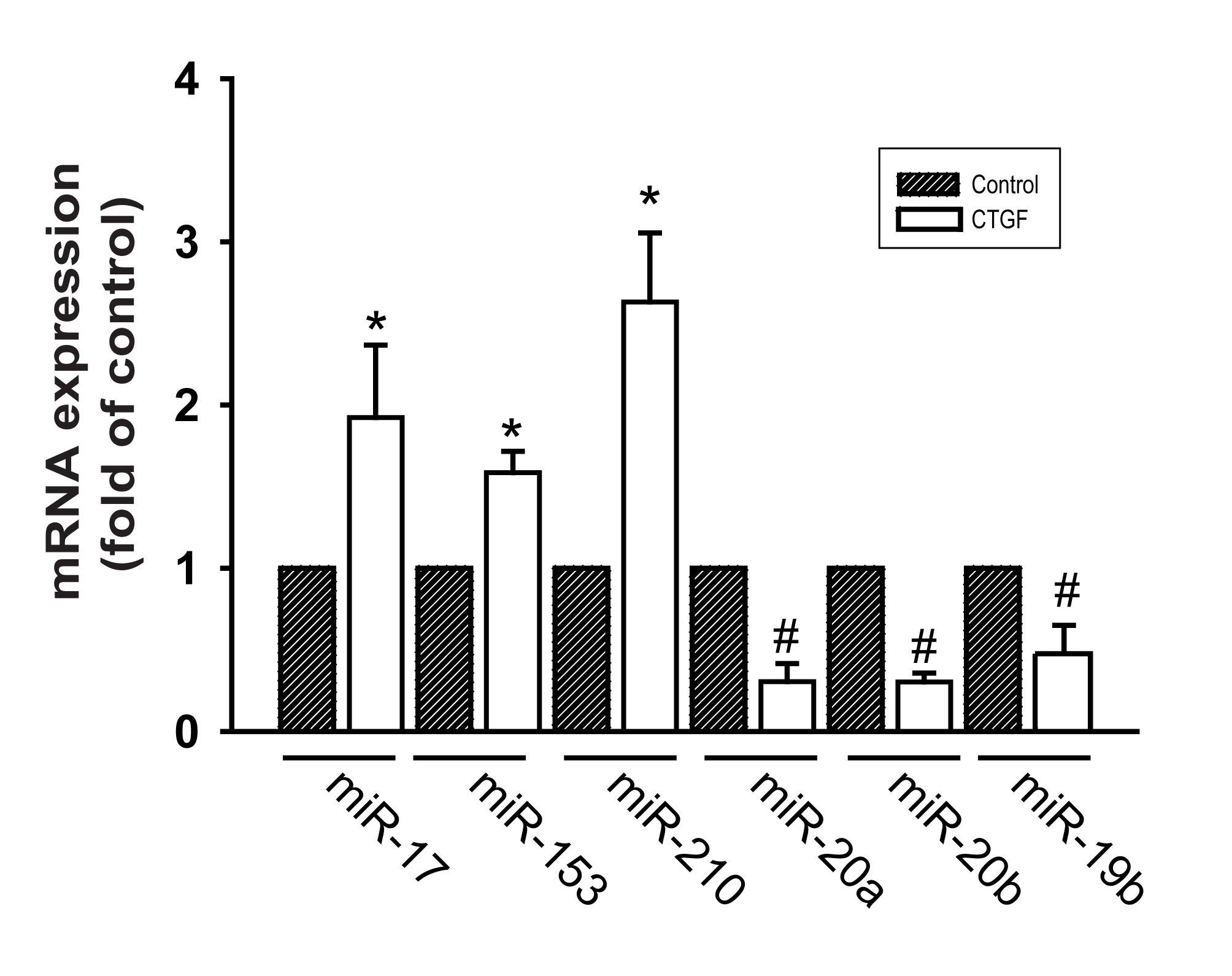
**

**Figure S1: The miRNAs expression after CTGF treatment in human OASFs.** The OASFs were incubated with CTGF (10 ng/mL) for 24 h, and miRNAs expression were examined by qPCR. Results are expressed as the mean ± S.E.M. *, *p* < 0.05 compared with control.


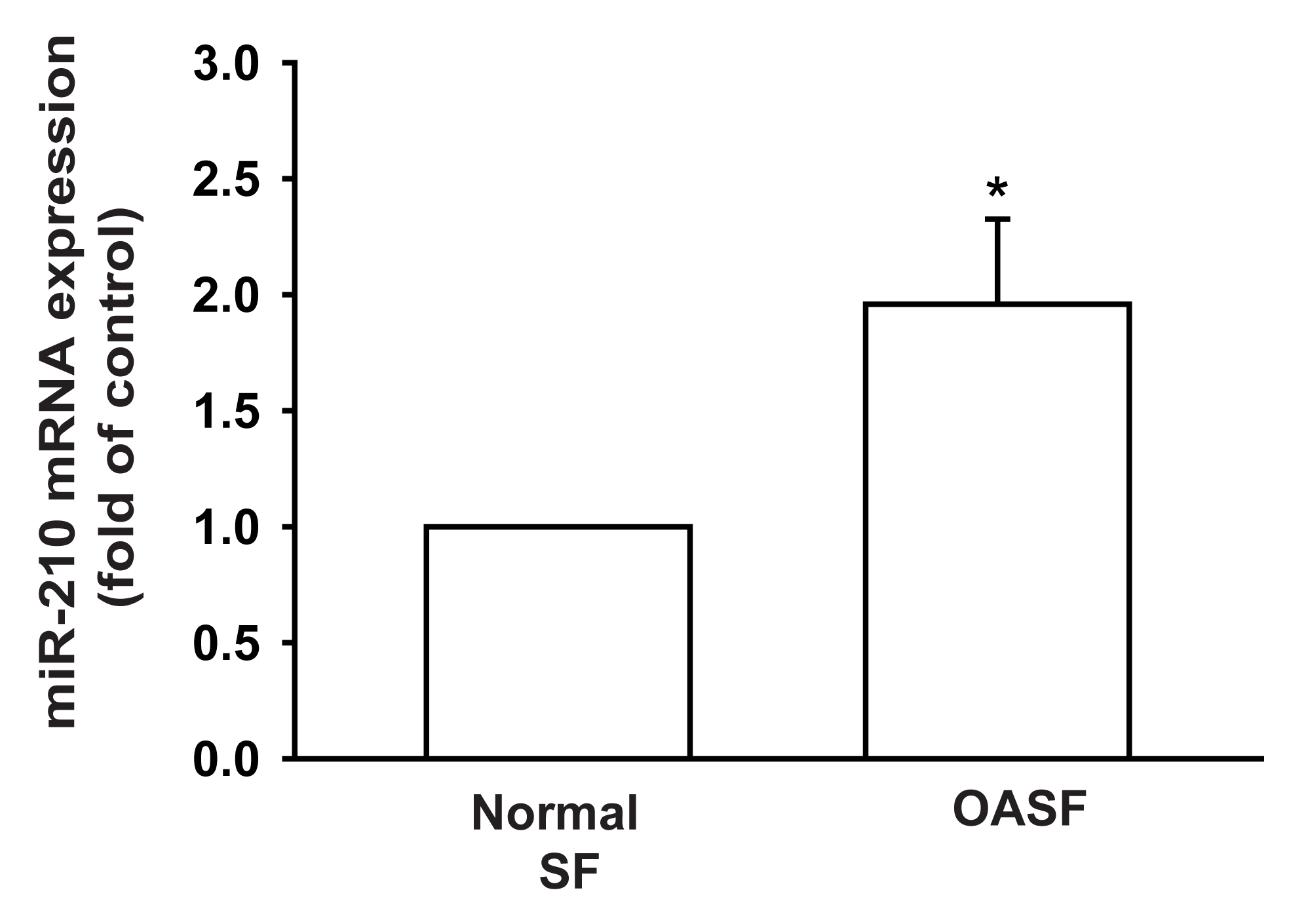


**Figure S2: The miR-210 expression in normal SF and OASF.** The miR-210 in normal SF and OASF were examined by qPCR. Results are expressed as the mean ± S.E.M. *, *p* < 0.05 compared with normal SF.

**
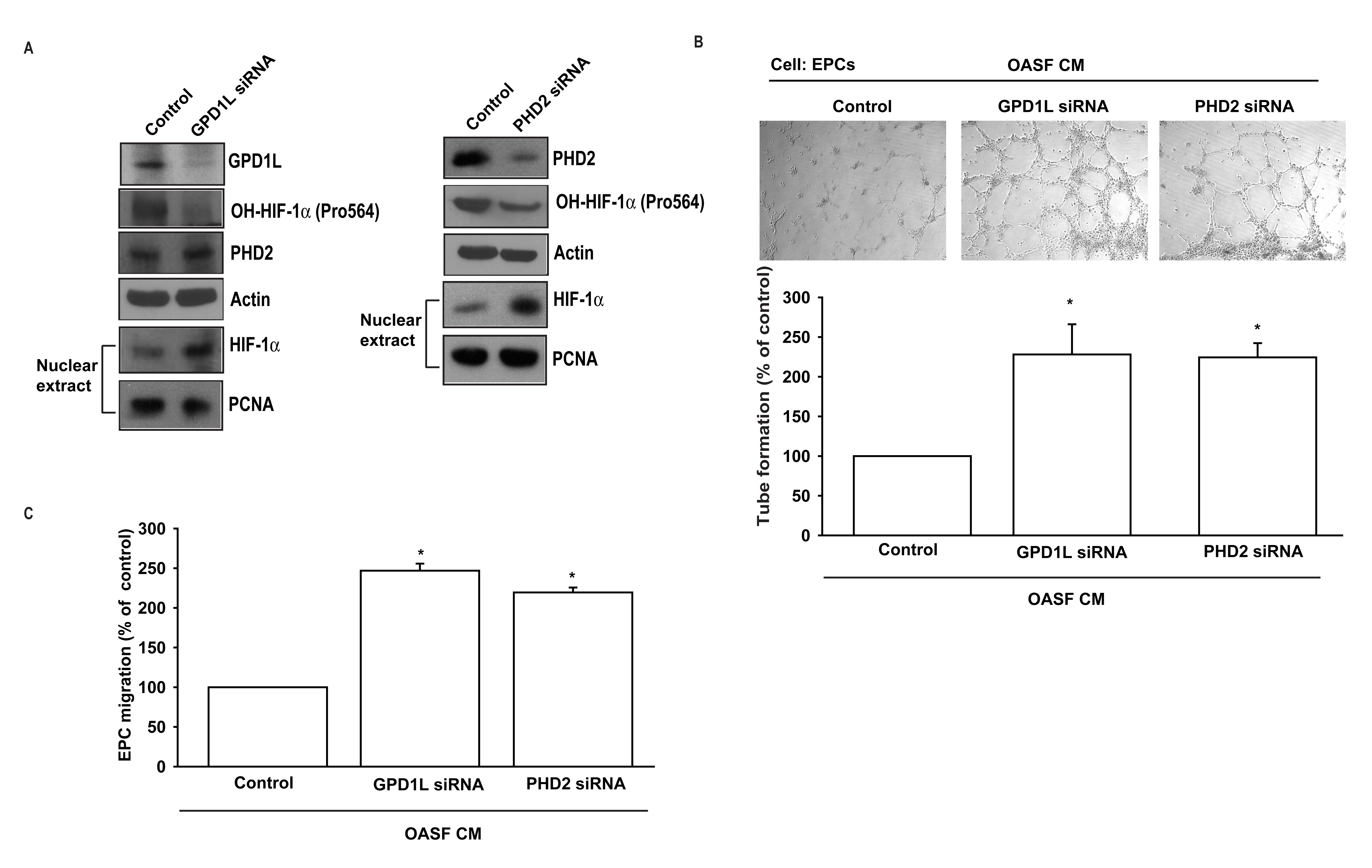
**

**Figure S3: GPD1L and PHD2 siRNA increases accumulation of HIF-1α into nucleus and angiogenesis in OASF.** (A) OASFs were transfected with GPD1L and PHD2 siRNA for 24 h. GPD1L, OH−HIF-1α (Pro564), PHD2, and HIF-1α expression derived by western blot. (B&C) Medium was collected as CM then applied to EPCs for 24 h. Cell capillary-like structure formation and migration in EPCs were examined by tube formation and Transwell assay. Results are expressed as the mean ± S.E.M. *, *p* < 0.05 compared with control.
